# TGF-β Signaling: New Insights Into Aortic Aneurysms

**DOI:** 10.1016/j.ebiom.2016.09.026

**Published:** 2016-10-03

**Authors:** Sean E. Thatcher

**Affiliations:** Graduate Center for Nutritional Sciences, Room 559, Charles T. Wethington Building, 900 S. Limestone, University of Kentucky, Lexington, KY 40536, USA

Aneurysm-osteoarthritis syndrome (AOS) is an autosomal dominant SMAD3 mutation that is characterized by aneurysms within the arterial tree and early-onset arthritis of the joints (van der Linde et al., 2012, van der Linde et al., 2013). It is uniquely different from Marfan's syndrome (MFS) which is a disorder of the extracellular matrix, specifically fibrillin-1 (Judge and Dietz, 2005). In this issue of *EBioMedicine*, van der Pluijm et al. dissect out the features of a whole body SMAD3 deficient mouse and compares it to a Fibulin-4 deficient mouse model (extracellular matrix defect model) (Fig. 8 of paper) (van der Pluijm et al., 2016). It is extremely interesting that while the phenotypes in aneurysm formation are similar, the molecular mechanisms are quite distinct. The authors note that there were no changes in aortic stiffness or MMP activity within the vascular smooth muscle cells (VSMCs) of the SMAD3-deficient mice, but instead more inflammatory infiltration of immune cells into the adventitia. Likewise, while both models show elevation of phosphorylated SMAD2 and ERK, the downstream targets of TGF-β signaling are decreased with SMAD3 deficiency, but have no effect or increased signaling with Fibulin-4 deficient mice (van der Pluijm et al., 2016).

Due to these differences in signaling, the authors suggest that blockade of inflammation may be a more appropriate treatment for those with SMAD3 dysfunction. Another recent study has also concluded that immune cells are dysfunctional with SMAD3 deficiency (Ye et al., 2013). Currently, there are no drug therapies for the treatment of aneurysms and surgery is the only option. Those that have MFS are typically placed on a beta-blocker, angiotensin converting enzyme inhibitor (ACEi) or an angiotensin receptor blocker (ARB) (Judge and Dietz, 2005, Habashi et al., 2006). Interestingly, those patients that have mutations within the TGF-β receptors (Loeys-Dietz syndrome, LDS), such as TGFBRI or TGFBRII, have elevated TGF-β signaling and may require medications that target this pathway or downstream targets (e.g. ERK), however this is still highly controversial (Wheeler et al., 2014, Akhurst and Hata, 2012, Cook et al., 2015). One of the problems with targeting TGF-β is it has both canonical and non-canonical pathways. The canonical pathway influences the phosphorylation of SMADs which can modulate gene expression patterns at both pre- and post-transcription within the cell. This pathway typically inhibits the proliferation of cells, however under SMAD3 deficiency, VSMCs were shown to have increased rates of proliferation (van der Pluijm et al., 2016). This further indicates that patients with SMAD3 mutations may not respond well to the current drug therapies that are used for MFS and LDS patients, and it may require further testing to determine what drug regiment might be best for this patient population.

AOS is also a recently identified aortic disease and is tied to familial inheritance (Wheeler et al., 2014). It is important for families to be screened for these mutations so that proper surveillance, via ultrasounds, can occur. Another interesting finding from the study by van der Pluijm et al., was the difference in survival rates between males and females with SMAD3 deficiency (van der Pluijm et al., 2016). Females tended to have better survival than males with SMAD3 deficiency, indicating that there may be gender differences. It has been well documented that abdominal aortic aneurysms (AAAs) are sexually dimorphic in both mice and humans with males also being more susceptible to the disease (Henriques et al., 2004). It would be interesting to see if this holds true in AOS patients as well.

This paper highlights the need for better understanding into the molecular signaling pathways that give rise to aneurysms. While under SMAD3 deficiency, TGF-β signaling is disrupted, it may require different types of drugs to help slow the progression of aneurysms in AOS patients. With the use of these novel mouse models, hopefully cardiovascular scientists will be able to bring new drugs and therapies to help patients live better and more productive lives.

## Disclosure

The author declared no conflicts of interest.
